# Identifying Novel Drug Targets for Epilepsy Through a Brain Transcriptome-Wide Association Study and Protein-Wide Association Study with Chemical-Gene-Interaction Analysis

**DOI:** 10.1007/s12035-023-03382-z

**Published:** 2023-05-29

**Authors:** Mengnan Lu, Ruoyang Feng, Chenglin Zhang, Yanfeng Xiao, Chunyan Yin

**Affiliations:** 1 Department of Pediatrics, The Second Affiliated Hospital of Xi’an Jiaotong University, Xi’an, 710054 Shanxi China; 2 Department of Joint Surgery, HongHui Hospital, Xi’an Jiaotong University, Xi’an, 710054 Shanxi China

**Keywords:** Epilepsy, Human brain proteome, Proteome-wide association studies, Transcriptome-wide association studies, Chemical-related gene set enrichment analysis

## Abstract

Epilepsy is a severe neurological condition affecting 50–65 million individuals worldwide that can lead to brain damage. Nevertheless, the etiology of epilepsy remains poorly understood. Meta-analyses of genome-wide association studies involving 15,212 epilepsy cases and 29,677 controls of the ILAE Consortium cohort were used to conduct transcriptome-wide association studies (TWAS) and protein-wide association studies (PWAS). Furthermore, a protein-protein interaction (PPI) network was generated using the STRING database, and significant epilepsy-susceptible genes were verified using chip data. Chemical-related gene set enrichment analysis (CGSEA) was performed to determine novel drug targets for epilepsy. TWAS analysis identified 21,170 genes, of which 58 were significant (*TWAS*_*fdr*_ < 0.05) in ten brain regions, and 16 differentially expressed genes were verified based on mRNA expression profiles. The PWAS identified 2249 genes, of which 2 were significant (*PWAS*_*fdr*_ < 0.05). Through chemical-gene set enrichment analysis, 287 environmental chemicals associated with epilepsy were identified. We identified five significant genes (*WIPF1*, *IQSEC1*, *JAM2*, *ICAM3*, and *ZNF143*) that had causal relationships with epilepsy. CGSEA identified 159 chemicals that were significantly correlated with epilepsy (*P*_*cgsea*_ < 0.05), such as pentobarbital, ketone bodies, and polychlorinated biphenyl. In summary, we performed TWAS, PWAS (for genetic factors), and CGSEA (for environmental factors) analyses and identified several epilepsy-associated genes and chemicals. The results of this study will contribute to our understanding of genetic and environmental factors for epilepsy and may predict novel drug targets.

**Supplementary Information:**

The online version contains supplementary material available at 10.1007/s12035-023-03382-z.

## Introduction

Epilepsy is a severe neurological condition characterized by repeated seizures that can lead to brain damage, disability, and even death [1]. Seizures are abnormal, excessive, or synchronous increases in neuronal activity, which can lead to behavioral manifestations [2]. Studies have shown that the lifetime prevalence of epilepsy is 7.60%, affecting 50–65 million individuals worldwide [3]. The etiology of epilepsy is unclear [4] and includes genetic, structural, infectious, metabolic, and immune factors. Moreover, the definitive cause cannot be determined in > 60% of patients [5]. Furthermore, current studies lack reliable human data from brain tissue or biomarker studies of common epilepsies [6] due to samples of human brain tissue being difficult to obtain [7]. Therefore, the etiology and pathogenesis of epilepsy must be further explored.

Genetic factors are one of the most important etiologies of epilepsy [8]. Genome-wide association studies (GWAS) for common epilepsy have identified genetic risk variants for generalized and focal epilepsy and febrile seizures [9]. Several candidate genes for epilepsy include calmodulin-regulated spectrin-associated protein 1-like protein 1 (*CAMSAP1L1*) [10], glutamate metabotropic receptor 3 (*GRM3*) [11], CD3 gamma subunit of T-cell receptor complex (*CD3G*), and solute carrier organic anion transporter family member 3A1 (*SLCO3A1*) [12]. However, it is difficult to identify genes that influence traits through GWAS alone [13]. Transcriptome-wide association study (TWAS) is a gene-based association approach that integrates GWAS data [14]. The International League Against Epilepsy Consortium on Complex Epilepsies (ILAE) has performed a TWAS with dorsolateral prefrontal cortex tissue, and identified 21 most likely biological epilepsy genes [15]. A TWAS has suggested three significant risk genes for epilepsy: tetratricopeptide repeat domain 21 B (*TTC21B*), *RP11-375N15.2*, and tankyrase (*TNKS*) [11]. These studies contributed to the understanding of the etiology and pathogenesis of epilepsy; however, they rarely explored the correlation between the brain proteome and epilepsy.

Previous studies have established an association between genotype and quantitative trait loci (QTL) on protein (pQTL) abundance and gene expression (eQTL) [16]. Proteome-wide association studies (PWAS) are based on the premise that causal variants in coding regions affect phenotypes by altering the biochemical functions of the gene’s protein products. In PWAS, variant-level associations are decomposed into individual gene values (determined by the association between variants and protein levels), and the combined association of these variants with outcomes is evaluated [17]. PWAS have been widely used to identify candidate genes in brain disorders such as Alzheimer’s disease [18], depression [19], stroke [20], and post-traumatic stress disorder [21]. Therefore, the possible pathogenesis of epilepsy can be explored from gene expression levels of proteins and transcriptomes using GWAS summary data.

**Fig. 1:**
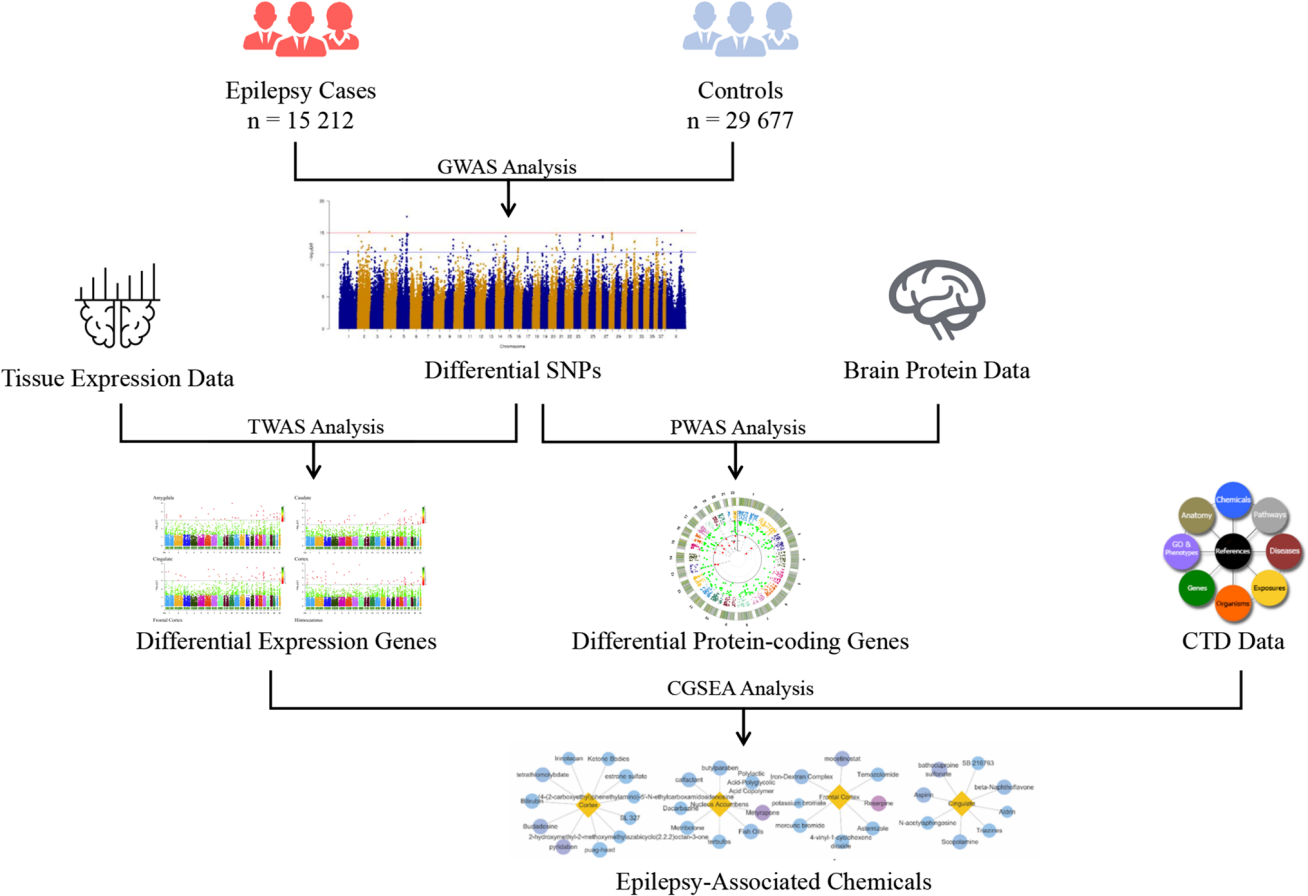
Flowchart of the experimental design. GWAS: genome-wide association studies; TWAS: transcriptome-wide association studies; CTD: Comparative Toxicogenomics Database; CGSEA: chemical-related gene set enrichment analysis

In this study, we aimed to determine the influence of genetic and environmental factors on epilepsy by performing TWAS and PWAS analyses based on a GWAS dataset. We investigated gene expression levels in the amygdala, caudate, cingulate, cortex, frontal cortex, hippocampus, hypothalamus, nucleus accumbens, putamen, and substantia nigra. Furthermore, we evaluated the relationship between genes identified by TWAS and PWAS and identified epilepsy-associated chemicals (Fig. 1).

## Methods and Materials

### Epilepsy GWAS Summary Data

The published GWAS summary data [15] for epilepsy used in our study was based on the International League Against Epilepsy (ILAE) launched by the Consortium on Complex Epilepsies. The analyzed data included 15, 212 cases (childhood absence epilepsy, juvenile absence epilepsy, juvenile myoclonic epilepsy, and generalized tonic-clonic seizures alone) and 29, 677 controls. This study is a large trans-ancestral GWAS meta-analysis that included not only Caucasian populations but also 531 and 147 cases from Asian and African populations, respectively. Single-nucleotide polymorphisms (SNPs) and genetic predictors of relative risk for all epilepsies were performed by using the PLINK-indep-pairwise command and PGA. Further information was detailed in a previously published study [15]. Ethical approval was not applicable to our study as publicly available data were used for all analyses.

### TWAS of Epilepsy

TWAS is a powerful method that integrates gene expression with GWAS to identify the genes associated with certain traits. Therefore, it is appropriate to study the genetic etiology of multiple phenotypes [22]. All genome-wide testing burdens were corrected to ensure the TWAS false positive rate was well-controlled in order to measure significant SNP-trait associations [23]. The software program, Functional Summary-based Imputation (FUSION, http://gusevlab.org/projects/fusion/), was used for TWAS and joint analyses of regions containing multiple significant associations [24]. Briefly, we calculated the reference weights of RnaSeq and human brain gene expression panels through the multiple prediction models running in FUSION, selected the best reference weights, unified the GWAS and reference SNPs, and removed/flipped alleles as appropriate. Each feature that expanded to 100,000 bp was defined as contiguous. The minimum *p*-value for including features in the model was 0.05. Features with *r*^2^ > 0.9 were considered identical, while features with *r*^2^ < 0.008 were considered independent. Then, we imputed GWAS *Z*-scores for any reference SNPs that were missing using the “ImpG” algorithm (https://github.com/huwenboshi/ImpG). Output columns included CHR (chromosome), P0 (gene start), P1, HSQ (heritability of the gene), BEST.GWAS.ID (rsID of the most significant GWAS SNP in locus), EQTL.R2 (cross-validation R2 of the best eQTL in the locus), MODELCV.R2 (cross-validation R2 of the best performing model), MODELCV.PV (cross-validation *P*-value of the best performing model), TWAS.Z (TWAS *Z*-score), and TWAS.P (TWAS *P*-value). After estimating the functional-GWAS association statistic, all features tested results were reported. All *P* values were then subjected to multiple testing corrections using the Benjamini–Hochberg procedure to gather *Q* values, which represent the minimum false discovery rate (FDR) threshold at which the contact is deemed significant [25]. FDR is less conservative than the Bonferroni approach and has greater ability (i.e., power) to find truly significant results [26]; thus, all *P* values are subjected to multiple testing correction using the FDR [27]. *TWAS*_*fdr*_ <0.05 was used as the significance threshold [28, 29].

High-frequency oscillations (HFOs, 80–500 Hz) recorded in intracranial electroencephalography have been regarded as neurobiomarkers for epileptogenic tissues [30]. HFOs can be identified not only in epileptic cerebral tissue but also in non-epileptic sites [31]. Thus, gene expression weights from many brain regions, including the amygdala, caudate, cingulate, cortex, frontal cortex, hippocampus, hypothalamus, nucleus accumbens, putamen, and substantia nigra, were used as references.

### PWAS of Epilepsy

Human brain proteome reference weight data were obtained using the Religious Orders Study and Rush Memory and Aging Project (ROS/MAP) and Banner Sun Health Research Institute (Banner) study data [32]. Individuals from the ROS/MAP and Banner were genotyped using the Affymetrix Precision Medicine Array following the manufacturer’s protocol, using the Qiagen GenePure kit to extract and purify DNA from brain cells. Genotyping was imputed to the 1000 Genome Project Phase 356 using the Michigan Imputation Server 57, and SNPs with imputation *R*^2^ > 0.3 were retained [19]. Genotyping was filtered to include only sites on the linkage disequilibrium reference panel provided by the FUSION pipeline. The epilepsy genetic effect (PWAS *z*-score) was calculated to evaluate the effects of significant SNPs in the GWAS on protein abundance. Finally, FUSION identified candidate genes that were associated with epilepsy that regulate protein abundance in the brain. The default settings and parameters recommended by FUSION were used for the analysis. All *P* values are subjected to multiple testing corrections using the FDR. *PWAS*_*fdr*_ <0.05 was used as the significance threshold.

### Gene Expression Validation of Epilepsy

Gene profiles (GSE88992) were downloaded from the Gene Expression Omnibus (GEO) database (https://www.ncbi.nlm.nih.gov/geo/). This study aimed to investigate the underlying molecular mechanisms in epilepsy. The epilepsy mouse model obtained by intrahippocampal microinjection of kainate (KA; 1 nmol/50 nL) was used in parallel with saline-injected animals as controls [33]. GSE88992 was downloaded from the GEO database using the GEO query package. Statistical analysis and visualization were performed using the R packages, “GEOquery” [34], “limma” [35], “ComplexHeatmap” [36], and “ggplot2.” Differentially expressed genes (DEGs) were identified based on adjusted *P* values. Veen plot was performed using the R packages “ggplot2.”

### Protein-Protein Interaction (PPI) Network and Transcription Factor (TF) Prediction

PPI networks and TFs were analyzed by using the STRING v11.5 database (STRING, https://string-db.org), which required a confidence score of 0.15 and “active interaction sources,” based on a previous study [28]. Cytoscape was used to visualize all interaction networks [29]. AnimalTFDB 4.0 database (http://bioinfo.life.hust.edu.cn) [37] and UCSC Genome Browser database (http://genome.ucsc.edu) [38] were used to predict TFs.

### Chemical-Related Gene Set Enrichment Analysis (CGSEA)

The chemical-gene expression annotation dataset used in this study was downloaded from the Comparative Toxicology Genomics Database (http://ctdbase.org/downloads/), which is a publicly available database that provides manually curated information about chemical–gene/protein interactions, chemical–disease, and gene–disease relationships [39]. CGSEA is a flexible tool for assessing the associations between chemicals and complex diseases. A detailed analysis method is provided in the original article [40]. In the present study, 5000 permutations were performed to obtain the empirical distribution of GSEA statistical data [41] for each chemical. Subsequently, the *p*-value of each chemical was calculated based on the empirical distribution of the CGSEA data. Based on previous studies [42], gene sets containing < 10 or > 200 genes were excluded to control for the influence of gene set size on the results. Chemicals with a *p*-value < 0.05 and absolute normalized enrichment score (NES) values of ≥ 1 were considered significantly enriched according to GSEA [43].

## Results

### TWAS Analysis of Epilepsy

**Fig. 2:**
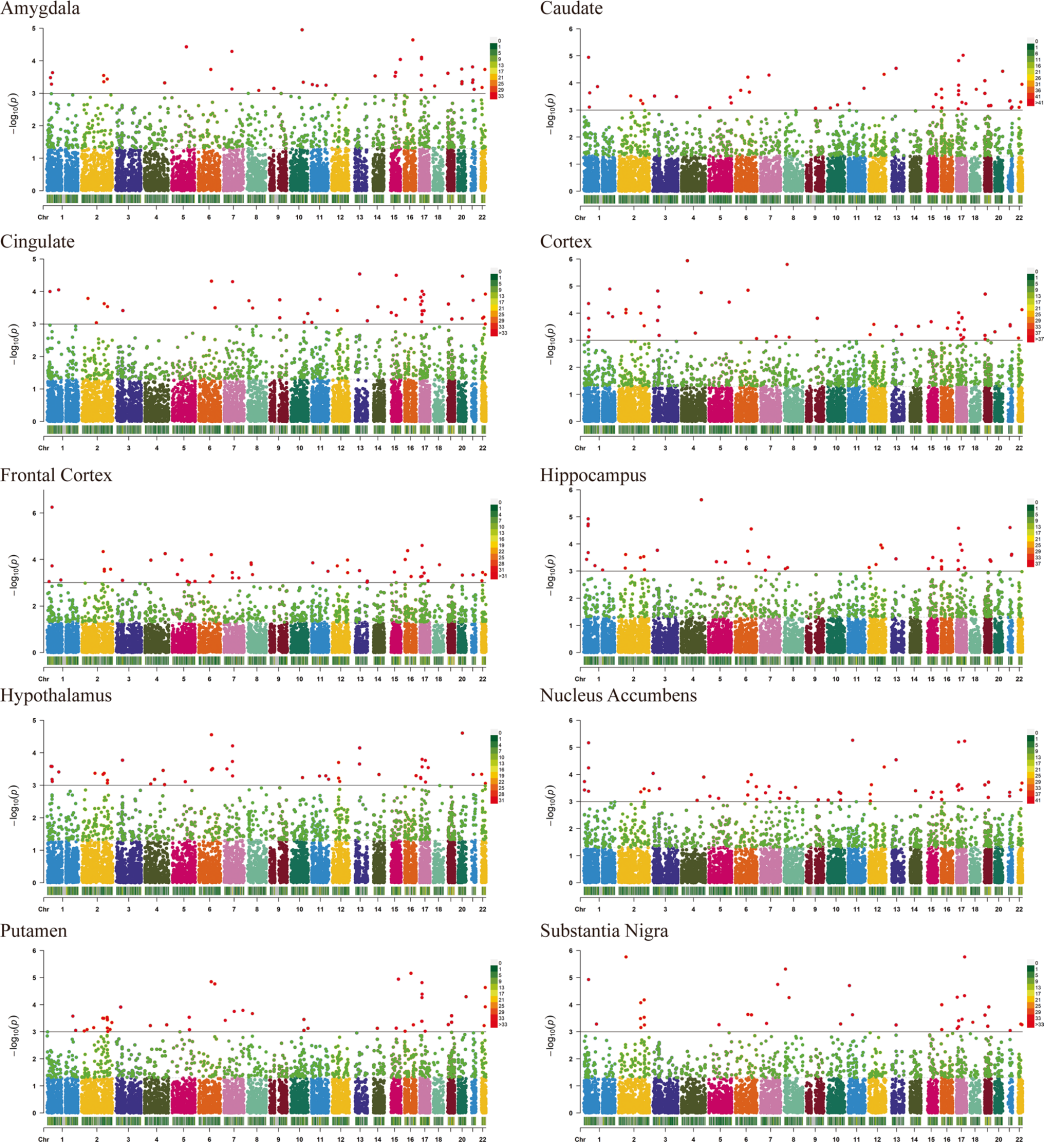
Manhattan plots from the epilepsy transcriptome-wide association study. The horizon line represents *P*_*twas*_ = 0.001. Each dot represents the genetically predicted expression of one specific gene for the amygdala, caudate, cingulate, cortex, frontal cortex, hippocampus, hypothalamus,nucleus accumbens, putamen, and substantia nigra. The *x* axis represents the genomic position of the corresponding gene, and the *y* axis represents the negative logarithm of the association *P*_*TWAS*_

TWAS analysis identified 21,170 genes from the epilepsy GWAS summary data (Fig. 2). After FDR correction, 58 unduplicated genes showed a significant association with epilepsy (*TWAS*_*fdr*_ < 0.05; Supplementary Information 1).

A comprehensive TWAS was performed to predict the relationship between epilepsy and significant genes found in the amygdala, caudate, cingulate, cortex, frontal cortex, hippocampus, hypothalamus, nucleus accumbens, putamen, and substantia nigra. These results are consistent with previous studies that have identified some risk loci for epilepsy [11, 15] (see Table 1).

**Table 1:** Replication of findings from previous studies

Gene	Chr	SNP	TWAS_*Z* score	TWAS_*p* value	CDE	Research
PNPO	17	rs4362431	4.8563	1.20E-06	Yes	ILAE
HSD3B7	16	rs2288005	5.1649	2.41E-07	Yes	ILAE
ZNF646	16	rs2288005	4.9236	8.50E-07	Yes	ILAE
VKORC1	16	rs2288005	−3.342	8.32E-04	Yes	ILAE
PRSS36	16	rs2288005	4.5402	5.62E-06	Yes	ILAE
ITGAX	16	rs1052352	−3.3345	8.54E-04	Yes	ILAE
FANCL	2	rs13032423	3.4440	5.73 E-04	No	ILAE
GABRA2	4	rs10517160	−3.7157	2.03 E-04	No	ILAE
TTC21B	2	rs6731869	-	< 0.05	Yes	Song et al.
TNKS	8	rs4841196	-	< 0.05	Yes	Song et al.
RP11-375N15.2	8	rs4841196	-	< 0.05	Yes	Song et al.

*Chr* chromosome; *SNP* single-nucleotide polymorphism; *TWAS* transcriptome-wide association study; *CDE* consistent direction effect, which is mainly determined by the positive and negative sign of TWAS_*Z* value; *ILAE* The International League Against Epilepsy Consortium on Complex Epilepsies; *Yes*: the sign of *z* values are consistent with our results; *No*: the sign of *z* values are not consistent with our results

### Common Genes Identified by TWAS and mRNA Expression Profiling

**Fig. 3:**
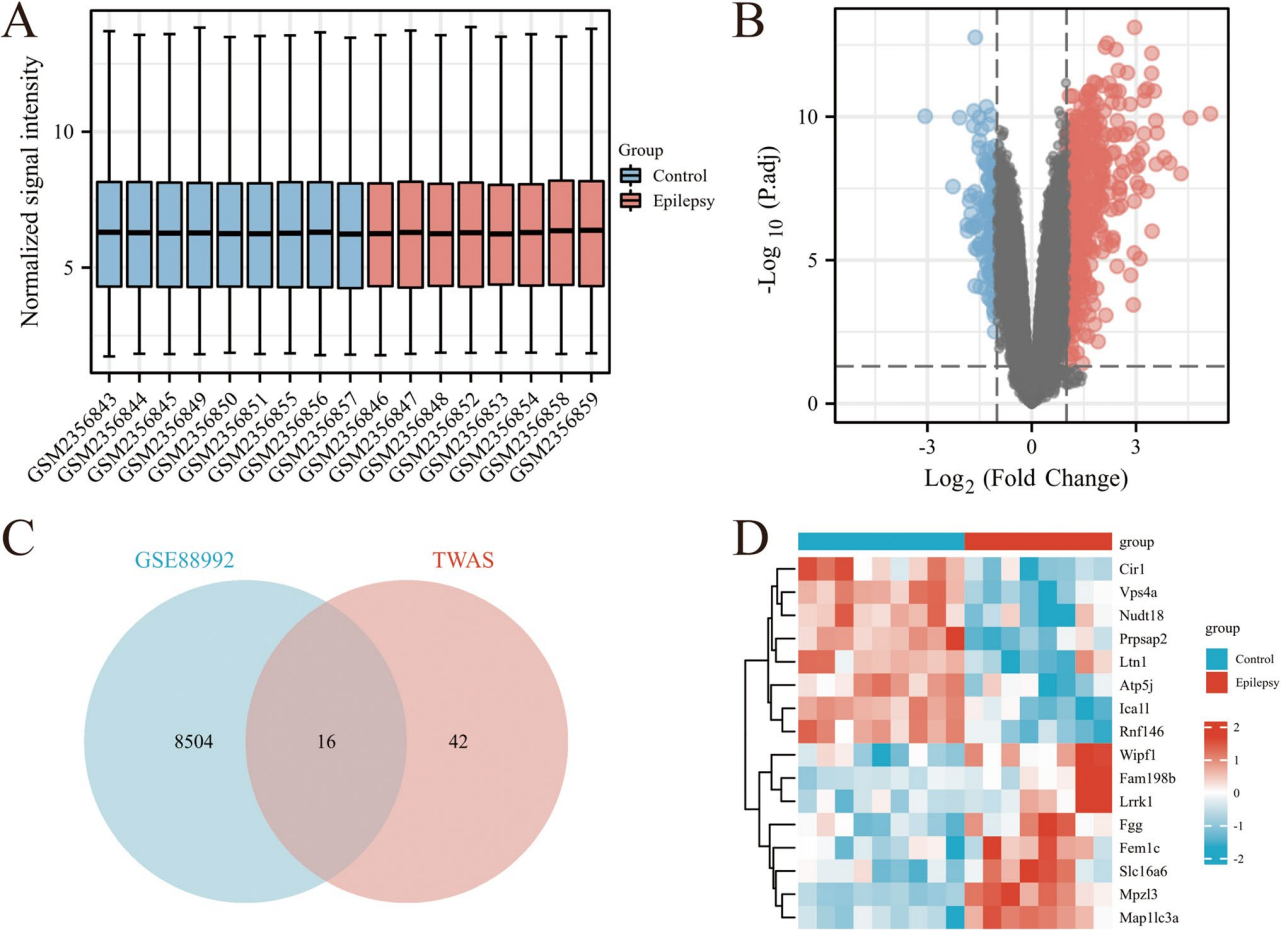
Gene expression profiles of epilepsy. **A** Normalized bar plot of the GSE88992 dataset. **B** Volcano plot of the GSE88992 dataset. **C** Venn diagram reveals the overlap of differentially expressed genes of GSE88992 and TWAS-identified genes. **D** Heatmap of 16 overlapping genes

To verify the reliability of the 58 TWAS-identified significant genes, we selected and analyzed the GEO dataset (GSE88992, Supplementary Information 2). GSE88992 was normalized and corrected (Fig. 3A). GSE88992 (Fig. 3B) contained 8520 genes and shared 16 common genes with TWAS-identified significant genes (Table 2, Fig. 3C). The expression of 16 overlapping genes are shown in Fig. 3D.

**Table 2:** Common genes identified by TWAS in conjunction with mRNA expression profiling

Gene	Chromosome	BEST.GWAS.ID	TWAS.Z	*TWAS.P*	*TWAS*_*fdr*_
ICA1L	2	rs6713203	3.56	3.65E-04	4.15E-02
CIR1	2	rs12994635	3.63	2.84E-04	4.04E-02
WIPF1	2	rs12994635	−3.51	4.43E-04	4.58E-02
FAM198B	4	rs12510198	−4.72	2.33E-06	2.15E-02
FGG	4	rs4392497	4.29	1.76E-05	3.16E-02
FEM1C	5	rs2034246	−4.12	3.73E-05	2.83E-02
RNF146	6	rs6902288	−4.19	2.80E-05	3.69E-02
NUDT18	8	rs7826924	4.80	1.58E-06	8.51E-03
MPZL3	11	rs2515800	−3.45	5.65E-04	4.28E-02
LRRK1	15	rs1807833	−3.91	9.15E-05	2.97E-02
VPS4A	16	rs11075734	−4.24	2.29E-05	2.60E-02
SLC16A6	17	rs12452511	4.53	5.93E-06	3.03E-02
PRPSAP2	17	rs9907723	−3.95	7.82E-05	3.56E-02
MAP1LC3A	20	rs6087626	−3.47	5.11E-04	4.31E-02
LTN1	21	rs11088088	3.55	3.90E-04	4.23E-02
ATP5J	21	rs4817045	3.78	1.55E-04	4.41E-02

### PWAS Analysis of Epilepsy

**Fig. 4:**
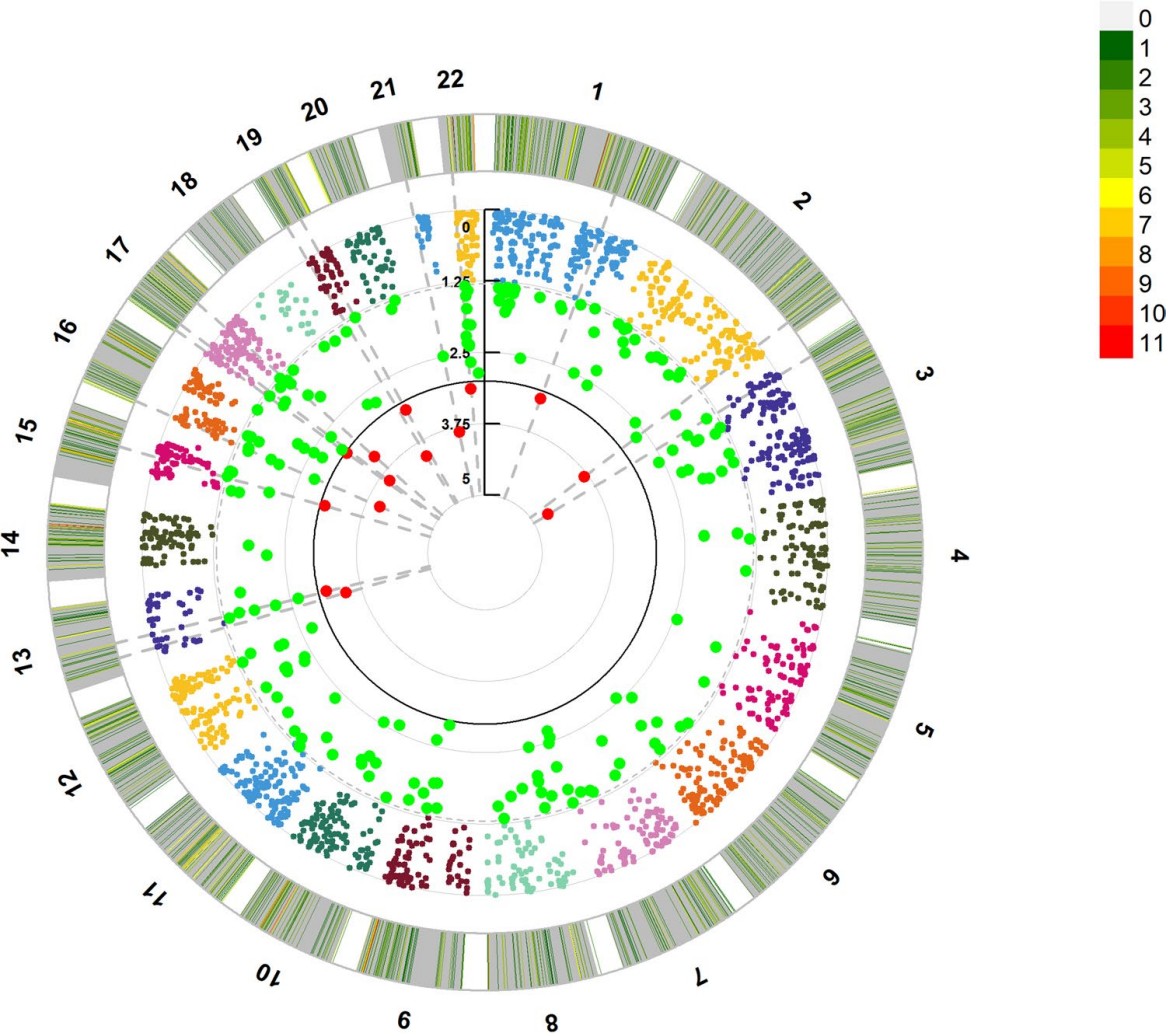
Manhattan plots from the epilepsy protein-wide association study. The horizon line represents *P*_*pwas*_ = 0.001. Each dot represents the genetically predicted expression of one specific protein-coding gene from the human brain. The *x* axis represents the genomic position of the corresponding gene, and the *y* axis represents the negative logarithm of the association *P*_*PWAS*_

PWAS analysis identified 2249 protein-coding genes from the epilepsy GWAS summary data (Fig. 4), wherein two protein-coding genes showed a significant association with epilepsy (*PWAS*_*fdr*_ < 0.05; Supplementary Information 3). These two epilepsy-susceptibility genes identified by PWAS were *IQSEC1* (IQ motif and Sec7 Domain ArfGEF 1, chromosome 3), and *JAM2* (junctional adhesion molecule 2, chromosome 21).

### Molecular Interaction of the Genes Identified by TWAS and PWAS

**Fig. 5:**
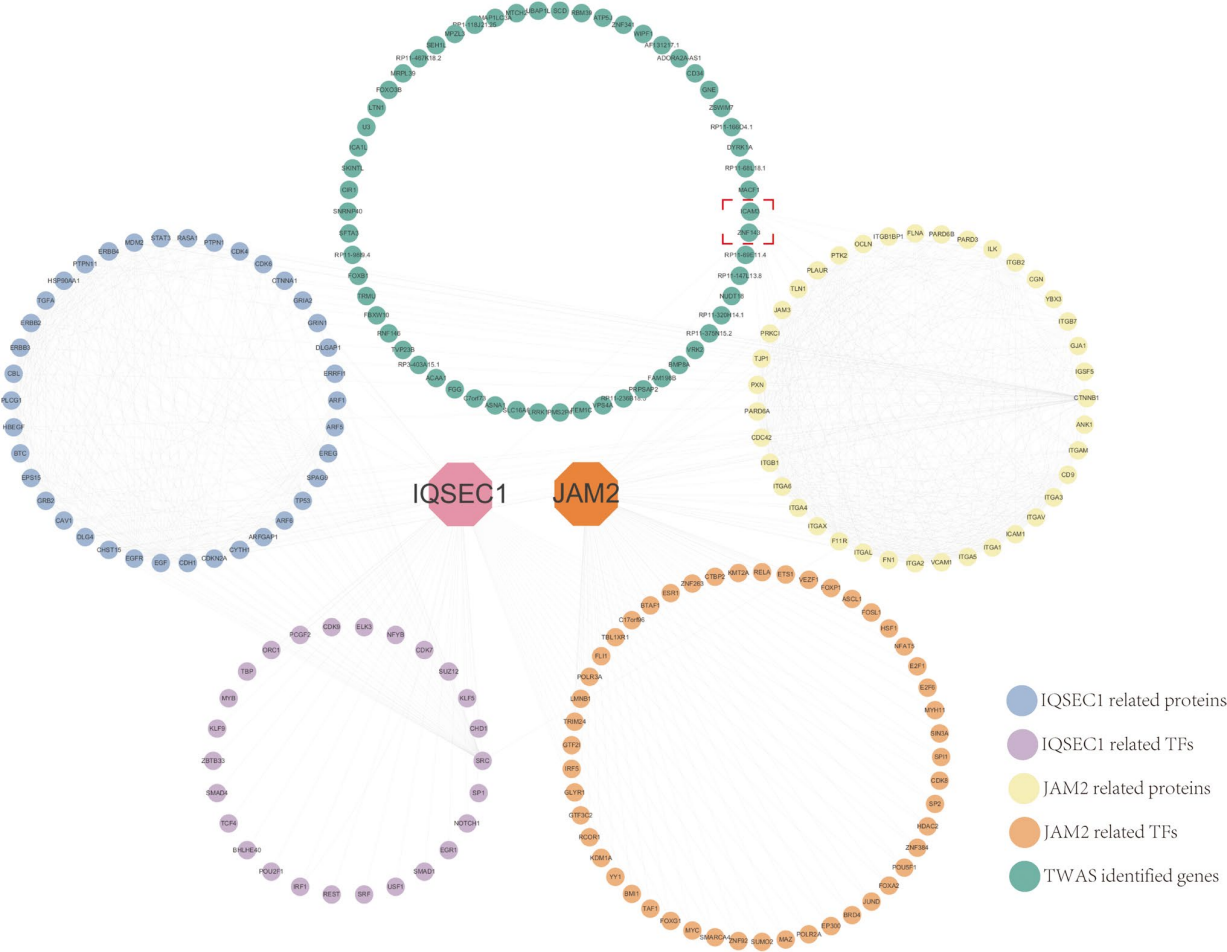
Relationship between TWAS and PWAS-identified genes in epilepsy

To verify the relationship between genes identified by TWAS and PWAS genes, we searched PWAS-identified gene-related proteins and TFs (Supplementary Information 4). Interestingly, *ICAM3* (intercellular adhesion molecule 3, chromosome 19) was observed to interact with *JAM2*. ZNF143 (zinc finger protein 143, chromosome 13) is predicted to be a TF common to both *IQSEC1* and *JAM2* (Fig. 5).

### CGSEA of the TWAS-Identified Genes

**Fig. 6:**
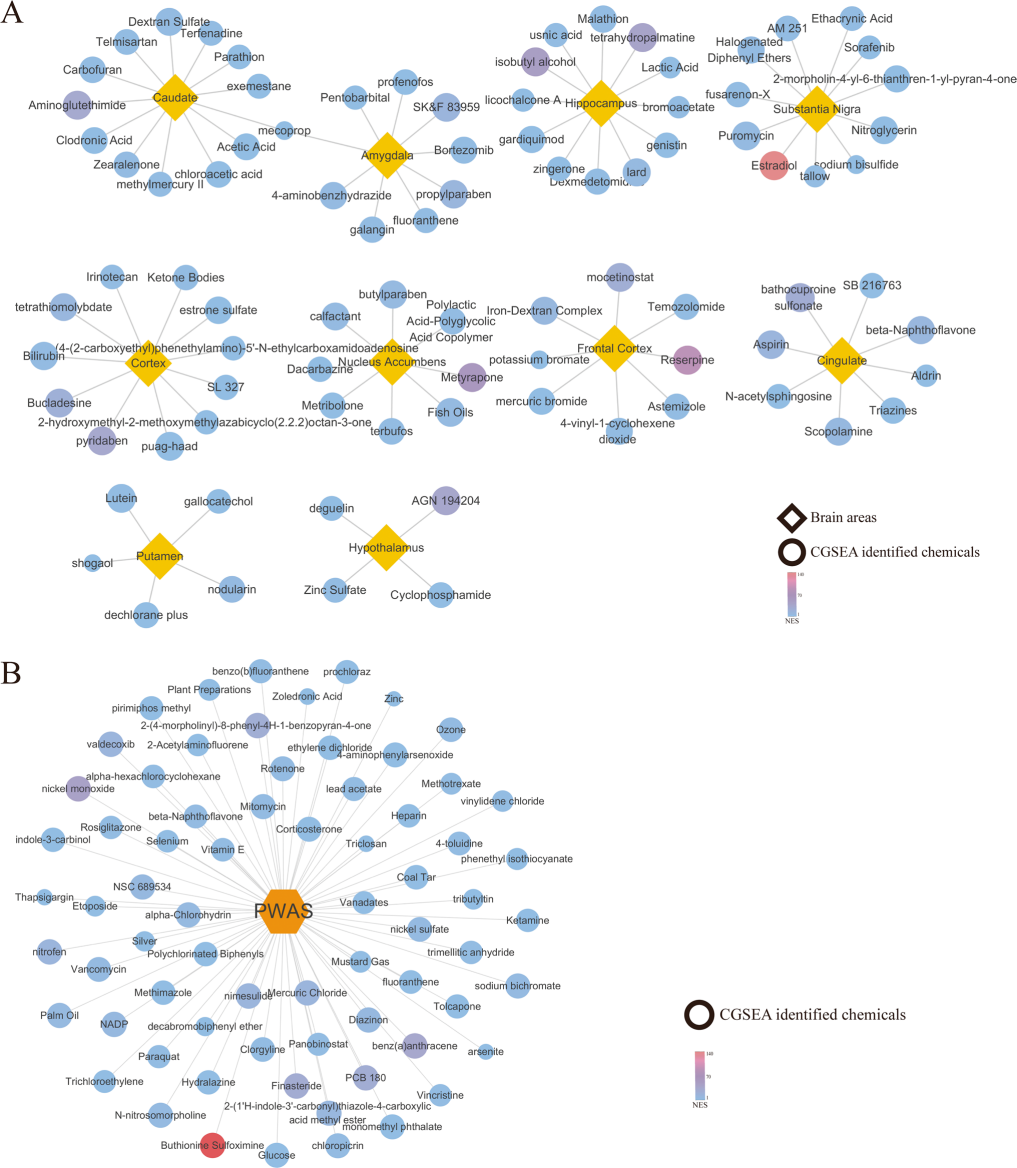
CGSEA results. **A** Network of chemicals and their areas of the brain based on TWAS-identified genes. **B** Network of chemicals based on PWAS-identified genes

CGSEA was performed to investigate the environmental factors that influence epilepsy. A total of 14,918 chemicals were identified, including 159 chemicals that correlated significantly with epilepsy (*P*_*CGSEA*_ < 0.05, |NES| ≥ 1, Supplementary Information 5). Figure 6 illustrates the constructed network of chemicals based on TWAS-identified and PWAS-identified genes.

## Discussion

Epilepsy has a higher incidence in children aged younger than 5 years than in those aged >5 years; moreover, children who present with epilepsy experience a high burden of cognitive and behavioral comorbidities [3]. Most seizures in patients with epilepsy arise from discrete pathological regions of abnormal brain tissue [44], including the frontal [45], temporal [46], occipital [47], and parietal lobes [48]. Thus, exploring as many brain regions as possible will provide new information for identifying biomarkers and understanding the etiology of epilepsy.

In this study, we identified several new candidate genes for epilepsy, such as *WIPF1*, *IQSEC1*, *JAM2*, *ICAM3*, and *ZNF143*. *WIPF1* (WAS/WASL interacting protein family member 1, related to endocytosis) has been identified by TWAS and mRNA expression profiles. An animal study demonstrated the potential significance of DNA methylation-dependent endocytosis regulation in the pathophysiology of temporal lobe epilepsy [49]. *IQSEC1* has been predicted to be involved in the regulation of postsynaptic neurotransmitter receptor internalization. Mutations of *IQSEC1* were found to be associated with intellectual disability [50], Alzheimer’s disease [51], and attention-deficit/hyperactivity disorder [52]. However, the potential role of *IQSEC1* in epilepsy has not been identified. Primary familial brain calcification (PFBC) is a neurodegenerative disorder characterized by calcium deposition in bilateral and symmetric brain [53]. Some PFBC patients would exhibit symptoms of neuropsychiatric symptoms, movement disorders, and epilepsy [54]. A case report showed Bi-allelic *JAM2* variants lead to learning difficulties and seizures [55]. Inflammatory cells, molecules, and associated pathways found in the nervous system and systemic tissues have been regarded as key factors in the development of epilepsy [56]. The intercellular adhesion molecule (ICAM) family is a subfamily of the immunoglobulin superfamily associated with immune responses, inflammation, and intracellular signaling [57]. *ICAM3* is a member of the ICAM family and can be expressed in gitter cells [58]. The neuronal protein Kidins220/ARMS, a protein that is mainly expressed in brain and neural cells, has been verified to coimmunoprecipitate with ICAM3 to regulate T-cell motility [59]. Therefore, we speculate that *ICAM3* may have a potential role in inflammation-related epilepsy, though further research is needed. Primary mitochondrial disease is a systemic disease, and seizures may be its presenting feature [60].

Prior studies have noted that mitochondrial oxidative stress and dysfunction have played a key role not only in epileptogenesis, but also result from seizures [61]. ZNF143 may protect cells from oxidative damage during mitochondrial dysfunction [62]. Cobalamin deficiency [63] and cobalamin-related remethylation disorder [64] may contribute to epilepsy. Whole-exome sequencing suggested mutations in ZNF143 are associated with inborn error of cobalamin metabolism [65]. These results implied the association between ZNF143 and epilepsy.

Antiseizure medications are the mainstay of treatment for patients with newly diagnosed epilepsy [66]. Although many antiepileptic drugs are available, approximately 30% of patients with epilepsy have uncontrolled seizures; thus, new therapeutic approaches must still be developed [67]. In this study, we extended the classic GSEA approach to detect the association between chemicals and epilepsy from our TWAS and PWAS results and identified 159 significantly enriched chemicals. Antiepileptic drugs [68] such as ketamine, corticosterone, pentobarbital, and ketone bodies were identified in this study. A ketogenic diet, particularly polyunsaturated fatty acids, is an effective treatment for refractory epilepsy [69] and may be beneficial for these patients [70]. This study confirms that polyunsaturated fatty acids (palm and fish oils) are associated with epilepsy, providing genetic evidence for the effects of a ketogenic diet on epilepsy. Vitamin E as a neuroprotective agent has been proven to be a useful therapeutic approach in epilepsy treatment [71]. Seizures can be manifestations of intracranial tumors [72]; thus, our results also identified some antineoplastic drugs, such as buthionine sulfoximine, methotrexate, vincristine, temozolomide, etoposide, and cyclophosphamide. Selenium has been suggested to be associated with epilepsy, which further supports the idea that selenium deficiency increases the risk of seizures, while supplementation may help alleviate seizure frequency and duration [73]. Genetic and environmental factors in early life are also important for neurodevelopment [74]. The etiology of neurodevelopmental disorders involves complex interactions between genes and the environment, which has been confirmed in schizophrenia [75] and autism [76]. Environmental chemical exposures in early life, including endocrine-disrupting chemicals (EDCs), influence health and disease susceptibility across the life course [77]. Multiple EDCs were identified in our study, including zearalenone, paraquat, trichloroethylene, polychlorinated biphenyls, and triclosan. Animal research has shown that polychlorinated biphenyl exposure increases the susceptibility to seizures in adulthood [78, 79]. EDCs have been widely elucidated as important risk factors for metabolic diseases such as diabetes and obesity [80]. Although EDCs are ubiquitous in the environment and affect human health [81], there are few published studies on the relationship between EDCs and epilepsy. Thus, more research is urgently needed to understand the mechanisms underlying the genetic predispositions and EDCs related to epilepsy.

Nevertheless, this study has some limitations. First, some genes related to epilepsy susceptibility identified here have not been verified by molecular biology experiments, which should be performed in future studies. Second, the pooled GWAS data are predominantly from European population and it is common to find type-I errors that are not under control for various reasons in GWAS analyses. Therefore, our results should be used with caution when studying other populations. Furthermore, some chemicals that were identified in this study have been previously demonstrated to play a role in epilepsy, whereas others have not yet been validated, which will require more clinical observations and cohort studies. However, to the best of our knowledge, this is the first large-scale study that used PWAS and CGSEA to identify candidate genes and chemicals related to epilepsy.

In this study, we aimed to determine the effects of genetic and environmental factors on epilepsy. Therefore, we performed TWAS and PWAS on epilepsy and identified multiple epilepsy-associated genes. We further performed CGSEA to identify multiple epilepsy-associated chemicals, including EDCs. Our results provide genetic evidence of the effects of EDCs on epilepsy. The results of this study expand our understanding of the genetic and environmental factors that affect epilepsy as well as potential novel drug targets.

## Supplementary Information

ESM 1 (XLSX 17774 kb)

ESM 2 (XLSX 5400 kb)

ESM 3 (XLSX 452 kb)

ESM 4 (XLSX 17433 kb)

ESM 5 (XLSX 785 kb)

## Data Availability

The datasets analyzed during the current study are available from the Database of Genomic Variants (http://projects.tcag.ca/variation/); the URLs for Consortia and Groups (https://www.preventcd.com); the BioGPS (http://biogps.gnf.org); the Gene Expression Omnibus database (https://www.ncbi.nlm.nih.gov/gds) (accession numbers GSE88992); and the UK biobank (http://geneatlas.roslin.ed.ac.uk/) (fields: 20002).
